# Self-reported practice patterns for cognitive preservation during whole-brain radiotherapy: a multinational cross-sectional survey of radiation oncologists in the Middle East and North Africa

**DOI:** 10.3389/fonc.2026.1927397

**Published:** 2026-10-13

**Authors:** Renda AlHabib, Fahad AlRowais, Issa Mohamad, Ali Hosni, Douaa Albukhari, Eman Eldebawy, Fatimah Kaabi, Majed Alghamdi, Mohammed Aldehaim, Saif Aljabab, Sara Owaidah, Shakir Al-Shakir, Ibrahim Alotain

**Affiliations:** 1 Radiation Oncology Department, King Fahad Specialist Hospital, Dammam, Saudi Arabia; 2 Radiation Oncology Department, King Hussein Cancer Center, Amman, Jordan; 3 Radiation Medicine Program, Princess Margaret Cancer Centre, University Health Network, University of Toronto, Toronto, ON, Canada; 4 Radiation Oncology, Princess Noorah Oncology Center, King Abdulaziz Medical City, Ministry of National Guard Health Affairs–Western Region, Jeddah, Saudi Arabia; 5 Radiation Oncology Department, National Cancer Institute, Cairo, Egypt; 6 College of Medicine, King Saud Bin Abdulaziz University for Health Sciences, Jeddah, Saudi Arabia; 7 Radiation Oncology Department, King Faisal Specialist Hospital and Research Centre, Riyadh, Saudi Arabia; 8 Radiation Oncology Department, King Saud University, Riyadh, Saudi Arabia

**Keywords:** brain metastases, hippocampal avoidance, memantine, neurocognitive preservation, whole-brain radiotherapy

## Abstract

**Background:**

Whole-brain radiotherapy (WBRT) remains an important treatment for patients with diffuse brain metastases, but radiation-induced cognitive decline is a major concern. Hippocampal-avoidance WBRT (HA-WBRT) and memantine are recommended by international guidelines for eligible patients, yet the extent of their adoption across the Middle East and North Africa (MENA) region remains unclear.

**Methods:**

A cross-sectional, anonymous questionnaire was distributed to radiation oncologists practising across the MENA region between January and March 2025 by email and professional social media. Because distribution was open, no sampling frame or response denominator could be defined, so the sample is a convenience sample. The instrument assessed self-reported practice patterns, availability and reported utilisation of HA-WBRT and memantine, perceived barriers, and follow-up and cognitive-assessment practices. All proportions are reported at the respondent level. Categorical comparisons used the chi-squared or Fisher’s exact test with exact p values and odds ratios; multivariable logistic regression with cluster-robust standard errors by centre was performed as an exploratory analysis. Reporting follows STROBE and CHERRIES.

**Results:**

In total, 124 radiation oncologists from 45 centres across 15 countries responded. HA-WBRT was reported as being routinely offered to eligible patients by 60 respondents (48.4%) and memantine by 34 (27.4%); 28 (22.6%) reported offering both routinely and 58 (46.8%) neither. When asked what proportion of their WBRT patients actually received both interventions, 70 (56.5%) answered never and only 4 (3.2%) more than 50%. Memantine was reported as available in the respondent’s centre by 48 (38.7%), while 66 (53.2%) did not know whether it was approved for brain metastases. The most frequently endorsed barriers were concern about missing intracranial disease (72; 58.1%) for HA-WBRT and lack of experience with the drug (84; 67.7%) for memantine. Formal cognitive testing was infrequent: 90 respondents (72.6%) reported using no cognitive assessment tool.

**Conclusion:**

Among the radiation oncologists who responded, self-reported routine use of cognitive-preservation strategies during WBRT was limited, with reported utilisation lower than reported offering. These findings describe the practices reported by a convenience sample and should not be interpreted as establishing the prevalence of underuse across the MENA region.

## 1 Introduction

Brain metastases represent a major clinical challenge in oncology, occurring in 30% to 40% of patients with solid tumours over the course of their disease. The incidence of these lesions is rising, driven by advancements in neuroimaging that allow for earlier detection and improved systemic therapies that prolong the lives of cancer patients (1, 2).

Whole-brain radiotherapy (WBRT) is a treatment modality historically utilised as the standard treatment for managing multiple brain metastases. Since the 1950s, it has been the most widely used treatment for these patients due to its effectiveness in palliation and in improving intracranial disease control (3–8).

However, as survival rates improve, the long-term neurocognitive toxicities associated with WBRT have become a primary concern for both clinicians and patients.

Radiation-induced cognitive decline may become clinically apparent within months after treatment and is characterised by significant deterioration in learning, episodic memory, executive function, and processing speed (9). Research into the pathogenesis of this decline has identified the exquisite radiosensitivity of neural stem cells located within the subgranular zone of the hippocampal dentate gyrus as an important contributing mechanism. These cells are vital for the formation of new memories, and preclinical models suggest that even modest doses of radiation can deplete this regenerative niche (10).

To mitigate these risks, several neuroprotective strategies have emerged. For patients with a limited number of brain metastases, landmark randomised trials such as N0574 and N107C have demonstrated that stereotactic radiosurgery provides better preservation of cognitive function and quality of life without compromising overall survival compared with whole-brain radiotherapy, and it is now generally preferred as the initial strategy in selected patients. Contemporary selection between stereotactic radiosurgery and WBRT is not determined by lesion number alone, but also by total intracranial tumour volume, lesion distribution, performance status, extracranial disease burden, anticipated survival, available systemic treatment options and local technical expertise (4, 11).

Despite this, WBRT remains a valid treatment option for patients with extensive or diffuse intracranial disease, including those with a high total lesion burden or leptomeningeal disease, in whom focal treatment is not considered appropriate.

In the setting where WBRT is required, pharmacological and technical interventions are now available to preserve cognition. In the phase III RTOG 0614 trial, memantine demonstrated a delay in time to cognitive decline and was well tolerated, with a toxicity profile comparable to placebo (12). Additionally, hippocampal-avoidance whole-brain radiotherapy (HA-WBRT) using intensity-modulated radiotherapy reduces hippocampal dose while preserving coverage of the remaining brain parenchyma. The phase II RTOG 0933 trial demonstrated improved memory and quality of life compared with historical controls (13). This was confirmed in the phase III NRG Oncology CC001 trial, where the addition of hippocampal avoidance to WBRT with memantine was associated with a 26% relative reduction in the risk of cognitive failure (14).

Based on this robust evidence, the ASCO–SNO–ASTRO guidelines now recommend that both memantine and hippocampal avoidance (HA) should be offered to patients planned for WBRT who meet the eligibility conditions specified in the guideline, namely a prognosis of at least four months and no metastases within the hippocampal avoidance region. These interventions are therefore conditionally, not universally, indicated (15).

Despite these clear recommendations, global adoption of these neuroprotective techniques remains heterogeneous. Patterns-of-care analyses in Europe and the United States reveal that while many radiation oncologists offer these techniques in principle, regular application is often limited. In a 2022 US survey, approximately 80% of respondents reported offering memantine and 73% offered HA-WBRT, marking a significant increase from earlier years (16).

Nevertheless, barriers to universal implementation persist, including resource-intensive treatment planning, insurance authorisation challenges, and clinician uncertainty regarding prognosis in patients with high intracranial disease burden. Resource availability may also influence adoption across different practice settings (17).

This study aims to describe patterns of care regarding the integration of neuroprotective strategies into the management of brain metastases among radiation oncologists who responded to a survey across the MENA region. By evaluating the reported offering and utilisation of HA-WBRT and memantine, this study seeks to characterise perceived clinical, technical, and institutional barriers to their implementation. It also evaluates reported follow-up and cognitive-assessment practices, with the aim of providing insight into current practice and potential areas for improvement.

## 2 Materials and methods

### 2.1 Study design and participants

This study employed a cross-sectional, questionnaire-based survey design using a non-probability convenience sampling approach. Invitations were directed at consultant-level radiation oncologists practising across the MENA region who are involved in the management of patients with brain metastases. Participation was voluntary and anonymous.

All participant characteristics — country of practice, centre, practice setting, years of independent practice, subspecialty involvement and involvement in the management of brain metastases — were self-reported. Consistent with the anonymous design, no identifying information was collected and no characteristic was independently verified; years of independent practice was the sole measure of professional seniority recorded.

The questionnaire was circulated through open professional channels, so no defined sampling frame existed and the number of potentially eligible clinicians reached could not be established. A response rate therefore cannot be calculated, the cohort is described throughout as a convenience sample, and no claim of regional representativeness is made. Reporting follows the STROBE statement for cross-sectional studies (22) and the CHERRIES checklist for web-based surveys (23).

The primary endpoint was self-reported practice patterns regarding the offering and utilisation of cognitive-preservation strategies, specifically hippocampal-avoidance whole-brain radiotherapy (HA-WBRT) and memantine, among patients receiving WBRT. Secondary endpoints were the factors associated with reported utilisation of HA-WBRT and memantine, clinical and institutional barriers to their implementation, and follow-up and cognitive-assessment practices after WBRT.

### 2.2 Survey development and distribution

A structured online questionnaire was developed using SurveyMonkey^®^ (SurveyMonkey Inc., San Mateo, California, USA). The instrument comprised 39 items, of which 36 collected analysable responses; an optional free-text name field, an institution city field and a free-text comment box were not analysed. Items were primarily single-choice and multiple-choice, with a small number of open-ended fields, and one item presented four clinical conditions in a fit/not-fit matrix. Survey domains included demographic and professional characteristics, current practice patterns in the management of brain metastases, utilisation of memantine and hippocampal-avoidance whole-brain radiotherapy (HA-WBRT), and perceived barriers to implementing these strategies. Questions regarding HA-WBRT referred specifically to its therapeutic use in patients with established brain metastases and did not address hippocampal avoidance during prophylactic cranial irradiation (PCI).

Item generation was informed by relevant published literature, by the ASCO–SNO–ASTRO guideline recommendations on cognitive preservation during WBRT (15), and by previously published patterns-of-care surveys in this field.

Prior to distribution, the questionnaire was pilot-tested by five consultant radiation oncologists, each with more than five years of clinical experience, to assess clarity, relevance, completeness and feasibility. On the basis of this feedback, the wording and phrasing of individual items were revised for grammatical clarity; no structural or content changes were required. No formal validation or reliability assessment was undertaken. The questionnaire was administered in English, and no translation or back-translation procedures were required. The complete questionnaire is provided as Supplementary Material.

For each item, respondents selected a single option unless otherwise stated. The multiple-response items were those addressing barriers to HA-WBRT, clinical situations considered unsuitable for HA-WBRT, and barriers to memantine use, for which respondents could endorse any number of options; percentages for these items therefore sum to more than 100.

Operational definitions. Throughout this report, “available” denotes that the respondent reported that memantine could be obtained within their centre; “approved” denotes that the respondent reported a formal institutional or regulatory approval for its use in brain metastases; “offered routinely” or “as a standard option” denotes that the respondent reported discussing the intervention with all eligible patients rather than only on request; and “reported utilisation” refers to the respondent’s estimate of the proportion of their own WBRT patients who actually received the intervention. These are distinct constructs and are reported separately; no equivalence between them is assumed. The questionnaire item itself was worded “routinely as a standard option for all patients receiving WBRT”. Because hippocampal avoidance and memantine are conditionally rather than universally indicated, responses to this item are interpreted throughout as routine offering to patients considered eligible for these interventions under current clinical recommendations, and not as their use in every patient receiving WBRT.

The survey was distributed between January and March 2025 through two channels: email invitations using the contact list of the Saudi Association of Radiation Oncology (SARO), and direct outreach and organic sharing on LinkedIn targeting radiation oncologists practising in the MENA region. The SARO contact list is not a formally audited membership roster; entries were added incrementally over time and include duplicates for individuals with more than one registered email address, so it could not be used to derive a reliable count of unique potentially eligible recipients. The number of individuals reached through LinkedIn could not be determined because of the open, non-controlled nature of the platform, which allows posts to be viewed and shared beyond the original target audience. Recruitment beyond Saudi Arabia relied primarily on the international reach of LinkedIn and on informal sharing among professional contacts, rather than on additional named national societies or institutional mailing lists.

An introductory page explained the study objectives and the estimated completion time, and confirmed that participation was voluntary and that responses were anonymous and confidential.

### 2.3 Data collection and ethical considerations

An optional free-text name field was included in the instrument but was not analysed or retained, and no other identifying information was collected. Responses were stored securely and were accessed only by the research team.

Relevant eligibility and demographic questions were mandatory, and only completed questionnaires were included in the final analysis; partially completed questionnaires were excluded in their entirety rather than analysed on a per-item basis. All 124 respondents therefore answered all 36 analysable items, and no item-level exclusions were necessary. The only incomplete field was the free-text institution name, which was left blank by 29 respondents (23.4%); this is an identifier rather than a survey response and does not affect any item-level denominator.

The SurveyMonkey collector was configured to disallow multiple responses, restricting each respondent to a single submission per device via a browser cookie. A unique link per individual recipient was not used, as the survey was distributed via a shared link rather than individualised invitations. In addition, responses were manually reviewed for potential duplicate submissions using available respondent information, including centre and country of practice; no duplicate responses were identified through this review.

The study was approved by the Institutional Review Board of King Fahad Specialist Hospital–Dammam, Saudi Arabia (IRB study number RAD0350; approval date 8 December 2024) following expedited review. Given the anonymous and voluntary nature of the survey, the IRB granted a waiver of consent documentation: participants were informed by means of an IRB-approved information sheet presented before the questionnaire, and consent was implied by completion. No written or signed consent form was therefore required. Because no identifiable personal data or images were collected, consent for publication of identifiable material is not applicable to this study. All procedures were conducted in accordance with the Declaration of Helsinki and with ethical standards for human research.

### 2.4 Statistical analysis

#### 2.4.1 Unit of analysis

The respondent, not the centre, is the unit of analysis throughout. Because more than one clinician responded from several institutions, respondent-level answers cannot be assumed to be statistically independent, and no attempt was made to reconcile or deduplicate responses to a single answer per centre. All proportions are therefore reported as the proportion of respondents, and statements such as “memantine was available in X% of centres” have been reworded accordingly. The institution field was free text and was left blank by 29 respondents (23.4%); among the 95 respondents who named an institution, entries mapped to 45 distinct centres after manual reconciliation of spelling and abbreviation variants, with 17 centres contributing more than one respondent and one centre contributing 11.

#### 2.4.2 Statistical analysis

Analyses were performed in Python 3.12 using pandas, SciPy and statsmodels. No target sample size was set and no formal sample-size calculation was performed; the analysis is based on all responses received during the recruitment window. The prespecified descriptive outcomes were the proportions of respondents reporting that they offer HA-WBRT and memantine routinely as a standard option. Categorical comparisons used the chi-squared test of independence, with Fisher’s exact test substituted where more than 20% of cells had an expected count below five; expected counts were inspected for every test and tests affected by sparse cells are identified as such. Exact two-sided p values are reported throughout, together with odds ratios and 95% confidence intervals for two-by-two comparisons. Multivariable logistic regression models were fitted for each of the two outcomes, entering practice setting, years of independent practice, neuro-oncology specialisation, country group, annual brain-metastasis volume and reported memantine availability; these models were refitted with cluster-robust standard errors grouped by self-reported centre to account for clustering, with respondents who did not name an institution treated as singleton clusters. No adjustment was made for multiple comparisons. Given the sample size, the absence of a defined sampling frame and the incompleteness of the centre identifier, all inferential analyses — including the Saudi Arabian subgroup analysis — are exploratory and hypothesis-generating, and the study is presented primarily as a descriptive report.

#### 2.4.3 Response recoding

Four non-standard entries were recoded before analysis, and no other data cleaning was performed. Two variant spellings of the “6–10 years” experience category were merged. On the item asking what proportion of WBRT patients received both interventions, one respondent answered “No”, which was treated as “never”. On the item asking what proportion received memantine, one response of “25–50%” was treated as “26–50%” and one response of “>50” as “>50%”. Each affects a single respondent; the first changes the reported “never” proportion for the combined-use item from 55.6% to 56.5%.

## 3 Results

### 3.1 Participant characteristics and practice patterns in brain metastases management

A total of 124 radiation oncologists from 45 centres across 15 countries completed the survey. Most respondents were male (76; 61.3%), and nearly half (61; 49.2%) had more than 10 years of independent practice. The majority (108; 87.1%) reported routine involvement in neuro-oncological practice. Respondents worked in academic or university centres (44; 35.5%), public or government hospitals (44; 35.5%), private practice (24; 19.4%) and military or charity settings (12; 9.7%). Saudi Arabia contributed the largest number of respondents (52; 41.9%), followed by Egypt (23; 18.5%), Jordan (11; 8.9%) and the United Arab Emirates (9; 7.3%). Sixty respondents (48.4%) reported treating 50 or more patients with brain metastases annually, while 85 (68.5%) reported delivering WBRT to fewer than 50 patients per year. Demographic and practice-related characteristics of the participants are summarised in **Table 1**.

**Table 1:** Demographic and practice characteristics of survey participants.

Demographic characteristics (N = 124 respondents)	N	%
Sex		
Male	76	61.3
Female	48	38.7
Years of independent practice		
1–5 years	35	28.2
6–10 years	28	22.6
>10 years	61	49.2
Practice setting		
Academic/University	44	35.5
Public/Government	44	35.5
Private practice	24	19.4
Military	11	8.9
Charity	1	0.8
Central nervous system specialist		
Yes	108	87.1
No	16	12.9
Country		
Saudi Arabia	52	41.9
Egypt	23	18.5
Jordan	11	8.9
United Arab Emirates	9	7.3
Tunisia	8	6.5
Other (Bahrain 3, Morocco 3, Türkiye 3, Oman 3, Kuwait 2, Algeria 2, Iraq 2, Qatar 1, Lebanon 1, Iran 1)	21	16.9

### 3.2 Utilisation of HA-WBRT and memantine

Offering and reported utilisation are presented separately below, because they are distinct constructs. Cross-tabulating the two offering items, 28 respondents (22.6%) reported offering both HA-WBRT and memantine routinely as a standard option, 32 (25.8%) reported offering HA-WBRT routinely without offering memantine routinely, 6 (4.8%) reported the converse, and 58 (46.8%) reported offering neither routinely. Reported utilisation was substantially lower: when asked what proportion of their WBRT patients actually received both HA-WBRT and memantine, 70 respondents (56.5%) answered “never”, 34 (27.4%) answered “less than 10%”, 8 (6.5%) “10–25%”, 8 (6.5%) “26–50%” and 4 (3.2%) “more than 50%”.

HA-WBRT was reported as being offered routinely as a standard option by 60 of 124 respondents (48.4%), while 64 (51.6%) reported offering it only when specifically requested. As set out in the Methods, this category denotes routine offering to patients considered eligible for hippocampal avoidance, rather than its use in every patient receiving WBRT.

Reported utilisation of HA-WBRT across all 124 respondents was as follows: 34 (27.4%) never used it, 44 (35.5%) used it in fewer than 10% of their WBRT patients, 17 (13.7%) in 10–25%, 11 (8.9%) in 26–50% and 18 (14.5%) in more than 50%. Among the 90 respondents who reported using HA-WBRT at all, 72 (80.0%) applied it to no more than half of their WBRT patients. Numerators and denominators are stated explicitly because these two denominators differ.

Memantine was reported as available in the respondent’s own centre by 48 respondents (38.7%). When asked whether it was approved for use in brain metastases, 46 (37.1%) answered yes, 12 (9.7%) answered no and 66 (53.2%) answered that they did not know; this “do not know” category is reported separately rather than combined with “no”. Memantine was reported as offered routinely as a standard option by 34 respondents (27.4%), whereas 90 (72.6%) offered it only when specifically requested. Reported utilisation was again lower: 85 respondents (68.5%) never gave memantine with WBRT, 32 (25.8%) gave it to fewer than 10% of patients, 2 (1.6%) to 10–25%, 2 (1.6%) to 26–50% and 3 (2.4%) to more than half.

When memantine was prescribed, it was most commonly initiated at the beginning of radiotherapy (28 respondents; 22.6%), with 8 (6.5%) starting it at the first clinic visit and 88 (71.0%) reporting that they did not prescribe it. On the separate item asking about intended treatment duration, 118 respondents (95.2%) selected “I do not prescribe memantine”, 4 (3.2%) selected six months, 1 (0.8%) three months and 1 (0.8%) an indefinite duration. These two items therefore give discordant counts of non-prescribers (88 and 118 respectively), indicating that some respondents interpreted one or both items differently; both are reported as observed, and the inconsistency is addressed in the Limitations.

Detailed utilisation patterns of HA-WBRT and memantine are summarised in **Table 2**.

**Table 2:** Utilisation patterns of HA-WBRT and memantine.

Practice and utilisation characteristics (N = 124 respondents)	N	%
Annual number of patients treated for brain metastases		
<50	64	51.6
50-100	44	35.5
101–200	12	9.7
>200	4	3.2
Annual number of patients treated with WBRT		
<50	85	68.5
50-100	28	22.6
101-200	8	6.5
>200	3	2.4
Is memantine available in your centre?		
Yes	48	38.7
No	76	61.3
Is memantine approved for brain metastases in your centre?		
Yes	46	37.1
No	12	9.7
I don’t know	66	53.2
Offer HA-WBRT (how the option is discussed with patients)		
Routinely as a standard option for all patients receiving WBRT^a^	60	48.4
Only when specifically asked	64	51.6
Offer memantine with WBRT (how the option is discussed with patients)		
Routinely as a standard option of management	34	27.4
Only when specifically asked	90	72.6
Reported proportion of own WBRT patients who received HA-WBRT		
Never	34	27.4
<10%	44	35.5
10-25%	17	13.7
26-50%	11	8.9
>50%	18	14.5
Reported proportion of own WBRT patients who received memantine		
Never	85	68.5
<10%	32	25.8
10-25%	2	1.6
26-50%	2	1.6
>50%	3	2.4
When do you start memantine?		
As soon as I see the patient in clinic	8	6.5
At the beginning of radiotherapy treatment	28	22.6
I don’t prescribe memantine	88	71.0
For how long do you prescribe memantine?		
3 months	1	0.8
6 months	4	3.2
Without time limitations	1	0.8
I don’t prescribe memantine	118	95.2

^a^Verbatim questionnaire wording. As explained in the Methods, responses to this item are interpreted as routine offering to patients considered eligible for hippocampal avoidance under current clinical recommendations, and not as the use of HA-WBRT in every patient receiving WBRT.

### 3.3 Barriers to implementation

The most frequently endorsed reasons for not offering HA-WBRT were concern about missing intracranial disease (72 of 124; 58.1%), resource-intensive treatment planning (66; 53.2%), delay in starting treatment (52; 41.9%), time constraints related to contouring (43; 34.7%), perceived insufficient evidence (34; 27.4%), technical difficulty with contouring (30; 24.2%), perceived absence of clinical benefit (20; 16.1%) and unavailability of the technique (16; 12.9%). Respondents could endorse more than one option, so percentages sum to more than 100 (**Figure 1**).

**Figure 1:**
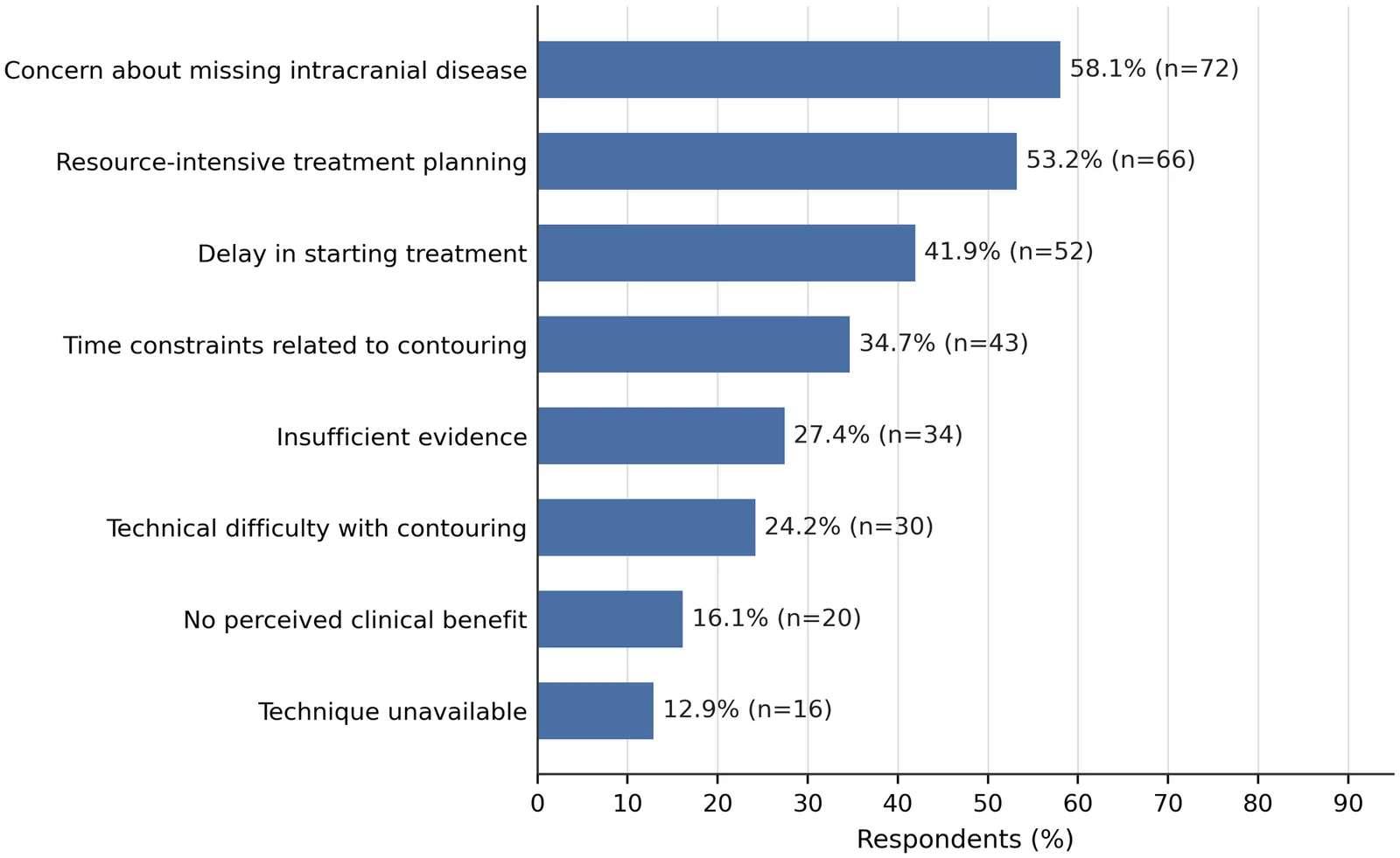
Reasons given by respondents for not offering HA-WBRT. All 124 respondents; multiple responses permitted, so percentages sum to more than 100. Concern about missing intracranial disease 72 (58.1%), resource-intensive treatment planning 66 (53.2%), delay in starting treatment 52 (41.9%), time constraints related to contouring 43 (34.7%), insufficient evidence 34 (27.4%), technical difficulty with contouring 30 (24.2%), no perceived clinical benefit 20 (16.1%), technique unavailable 16 (12.9%). HA-WBRT, hippocampal-avoidance whole-brain radiotherapy.

The clinical situations most often considered unsuitable for HA-WBRT were leptomeningeal disease (102 of 124; 82.3%), poor performance status (100; 80.6%), small cell lung cancer (SCLC) histology (47; 37.9%) and older age (44; 35.5%). Respondents could endorse more than one option (**Figure 2**).

**Figure 2:**
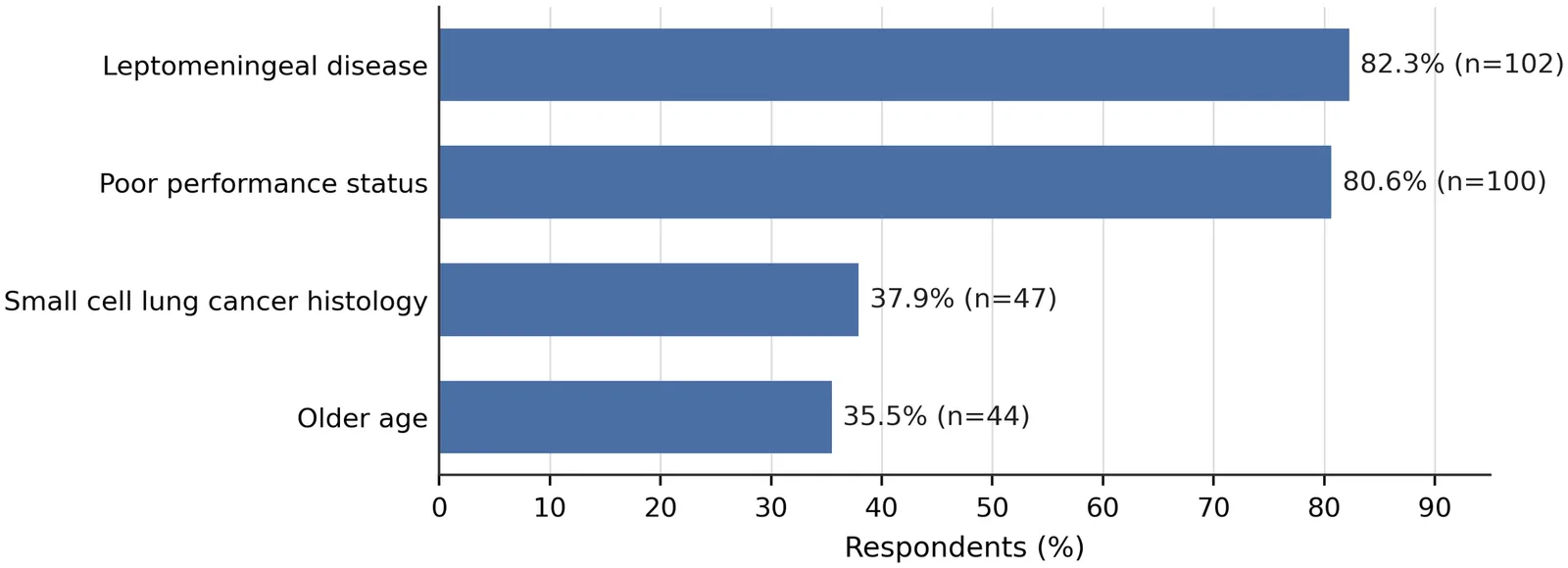
Clinical situations in which respondents considered patients unsuitable for HA-WBRT. All 124 respondents; multiple responses permitted, so percentages sum to more than 100. Leptomeningeal disease 102 (82.3%), poor performance status 100 (80.6%), small cell lung cancer histology 47 (37.9%), older age 44 (35.5%).

The most frequently endorsed barriers to memantine use were lack of experience with the drug (84 of 124; 67.7%), perceived insufficient evidence (59; 47.6%), reimbursement difficulties related to off-label use (58; 46.8%), concerns about patient compliance (55; 44.4%), side-effect and safety concerns (48; 38.7%) and perceived absence of clinical benefit (25; 20.2%). Respondents could endorse more than one option (**Figure 3**).

**Figure 3:**
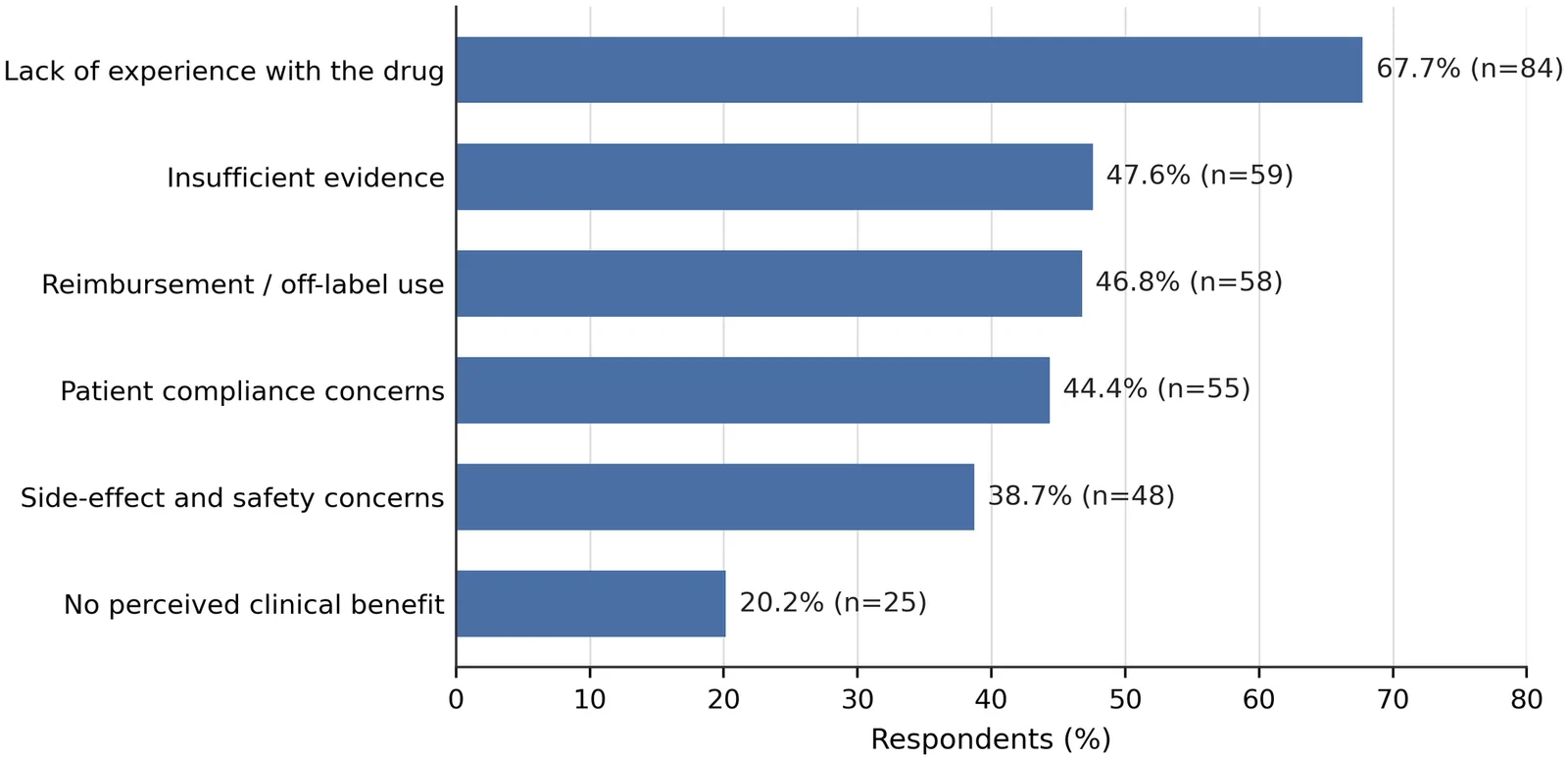
Reported barriers to the use of memantine with whole-brain radiotherapy. All 124 respondents; multiple responses permitted, so percentages sum to more than 100. Lack of experience with the drug 84 (67.7%), insufficient evidence 59 (47.6%), reimbursement or off-label use 58 (46.8%), patient compliance concerns 55 (44.4%), side-effect and safety concerns 48 (38.7%), no perceived clinical benefit 25 (20.2%).

### 3.4 Follow-up and cognitive assessment practices

On the item asking what type of follow-up was performed, 54 respondents (43.5%) reported no follow-up. Where follow-up was undertaken it most often combined brain imaging with clinical assessment (27; 21.8%) or consisted of brain imaging alone (21; 16.9%). Formal cognitive testing was infrequent: 90 respondents (72.6%) reported using no cognitive assessment tool, while the Mini-Mental State Examination was used by 22 (17.7%), the Hopkins Verbal Learning Test by 6 (4.8%) and other approaches by 6 (4.8%). Cognitive assessment, when performed, was carried out before radiotherapy by 22 respondents (17.7%) and during follow-up by 22 (17.7%).

Follow-up strategies and cognitive assessment practices are summarised in **Table 3**.

**Table 3:** Follow-up and cognitive assessment practices.

Follow-up and cognitive assessment (N = 124 respondents)	N	%
What type of follow up?		
Clinical assessment only	8	6.5
Use of cognitive tests	2	1.6
Brain imaging	21	16.9
Brain imaging + clinical assessment + cognitive tests	7	5.6
Brain imaging + clinical assessment	27	21.8
Brain imaging + cognitive tests	5	4.0
No follow up	54	43.5
Which test do you typically use for cognitive assessment?		
Mini-Mental State Examination	22	17.7
Hopkins Verbal Learning Test	6	4.8
Other (please specify)	6	4.8
I don’t use any test	90	72.6
When do you perform cognitive assessment?		
Prior to radiotherapy	22	17.7
During follow up	22	17.7
Upon completion of radiotherapy	2	1.6
I don’t use cognitive tests	78	62.9

### 3.5 Subgroup analysis: Saudi Arabia

A *post hoc* subgroup analysis was undertaken for the 52 respondents based in Saudi Arabia, drawn from 16 distinct self-reported centres — the only country with a sample large enough to permit any within-country comparison. This analysis was not prespecified and is exploratory. Routine offering of HA-WBRT was reported by 25 respondents (48.1%) and of memantine by 20 (38.5%). Considering the two strategies together, 16 respondents (30.8%) offered both routinely, 23 (44.2%) offered neither, 9 (17.3%) offered HA-WBRT only and 4 (7.7%) memantine only.

All 5 respondents working in private centres reported offering HA-WBRT routinely, compared with 20 of 47 (42.6%) in academic, public, military or charity settings (Fisher’s exact p = 0.020). Because one cell is zero, no finite odds ratio or confidence interval can be estimated for this comparison, and the result rests on five respondents. Years of independent practice was associated with routine HA-WBRT offering in this subgroup (χ² = 6.48, df = 2, p = 0.039), reported by 13 of 19 respondents (68.4%) in practice for 1–5 years, 3 of 13 (23.1%) for 6–10 years and 9 of 20 (45.0%) for more than 10 years. Neuro-oncology specialisation was not associated with HA-WBRT use in this subgroup (Fisher’s exact p = 0.205).

### 3.6 Exploratory associations

In the whole cohort, reported availability of memantine in the respondent’s centre was associated with routine offering of memantine (24 of 48 versus 10 of 76; Fisher’s exact p < 0.001; OR 6.60, 95% CI 2.76–15.80) and with routine offering of HA-WBRT (30 of 48 versus 30 of 76; Fisher’s exact p = 0.016; OR 2.56, 95% CI 1.21–5.38). Practice setting was associated with routine HA-WBRT offering (χ² = 21.00, df = 3, p < 0.001), driven by private practice, where 21 of 24 respondents (87.5%) reported routine offering. Years of independent practice (p = 0.412), neuro-oncology specialisation (p = 0.427) and country group (p = 1.000) were not associated with routine HA-WBRT offering.

In multivariable logistic regression, private practice setting (OR 15.31, 95% CI 3.73–62.83, p < 0.001, reference academic) and reported memantine availability (OR 4.56, 95% CI 1.67–12.42, p = 0.003) were independently associated with routine offering of HA-WBRT; the model pseudo-R² was 0.198. For routine offering of memantine, reported availability was the dominant predictor (OR 6.86, 95% CI 2.45–19.24, p < 0.001), with private practice reaching nominal significance (OR 3.86, 95% CI 1.04–14.30, p = 0.043). After refitting with cluster-robust standard errors grouped by self-reported centre, the associations with private practice (OR 15.31, 95% CI 3.28–71.42, p = 0.001) and memantine availability (OR 4.56, 95% CI 1.39–14.97, p = 0.012) persisted for HA-WBRT, and memantine availability persisted for memantine (OR 6.86, 95% CI 2.16–21.79, p = 0.001), whereas the association between private practice and memantine offering was attenuated and no longer significant (OR 3.86, 95% CI 0.81–18.38, p = 0.089). Expected cell counts were inspected for every test. No correction was made for multiple comparisons and all of these analyses should be interpreted as exploratory.

## 4 Discussion

The findings of this pattern-of-care analysis reveal a significant gap between international clinical guidelines and the routine practice of radiation oncologists across the Middle East and North Africa (MENA) region. While the ASCO–SNO–ASTRO guidelines recommend that both hippocampal avoidance and memantine be offered to patients planned for WBRT who meet the specified eligibility conditions — an expected survival of at least four months and no metastases within the hippocampal avoidance region — our study found that 22.6% of respondents reported offering both routinely, and that reported utilisation was lower still: 56.5% said that none of their WBRT patients actually received both interventions. Reported offering in this cohort is lower than in recent reports from the United States, where surveys indicate that roughly 63% of radiation oncologists offer both (16). Because the two surveys used different question wording and different denominators, this comparison is indicative rather than direct.

Routine offering of HA-WBRT was reported by 48.4% of respondents, although reported utilisation was considerably lower, with 27.4% reporting that no WBRT patient received it. Awareness was not measured directly in this survey and cannot be inferred from self-reported offering. This contrasts with a European pattern-of-care analysis, which reported that 85% of radiation oncologists offer hippocampal sparing in principle, but only 25% use it regularly (17).

The most frequently endorsed barrier in our study was concern about missing intracranial disease (58.1%). Although the NRG Oncology CC001 trial found that recurrence within the hippocampal avoidance region is uncommon (14, 18), this concern remained the single most commonly cited reason for not offering HA-WBRT among our respondents, suggesting that the perceived risk exceeds the reported risk. Furthermore, the resource-intensive nature of treatment planning (53.2%) and treatment delays (41.9%) were reported as significant challenges. These findings align with both US and European data, where limited staff capacity and complex intensity-modulated radiotherapy planning were frequently reported obstacles (16). In our exploratory Saudi Arabian subgroup analysis, all five respondents in private practice reported offering HA-WBRT routinely, compared with 42.6% of respondents in other settings (Fisher’s exact p = 0.020); the finding rests on five respondents and should be regarded as hypothesis-generating only.

The reasons provided by radiation oncologists for avoiding HA-WBRT — specifically leptomeningeal disease (82.3%), poor performance status (80.6%), and SCLC histology (37.9%) — are largely justified by current evidentiary limits. Patients with leptomeningeal disease are traditionally excluded from HA trials due to the high risk of recurrence in the CSF-rich regions near the hippocampus (14). Furthermore, the guideline conditions these recommendations on an expected survival of at least four months (15). The questionnaire item used the general term “poor performance status” and did not specify a Karnofsky Performance Status threshold, so responses to this item cannot be equated with any particular numerical cut-off. Lastly, the NRG CC001 trial excluded patients with SCLC (18). Although the PREMER and NRG-CC003 trials have since strengthened the case for hippocampal avoidance during prophylactic cranial irradiation in SCLC (20, 21), evidence from the prophylactic setting does not translate directly to the therapeutic setting, and the role of therapeutic HA-WBRT in patients with established SCLC brain metastases remains unresolved.

The underutilisation of memantine in our study — routinely prescribed by only 27.4% of respondents — is similar to findings from the European ESTRO survey (30%) but contrasts sharply with the US (80%) (16, 17).

The leading reason for non-adoption was a lack of experience with the drug (67.7%). One possible contributor to this experience gap is that the primary endpoint of the landmark RTOG 0614 trial (delayed recall at 24 weeks) did not reach conventional statistical significance (p = 0.059), although the trial was underpowered because of early patient loss (12). This survey did not ask respondents about their awareness of that result, so the connection is a hypothesis rather than a finding. Furthermore, our findings regarding reimbursement issues (46.8%) as a barrier to memantine use align with international studies where insurance authorisation and the “off-label” perception of neuroprotective agents remain problematic. Interestingly, Lamba et al. reported population-based prescription rates of memantine as low as 2.2% to 5.14% in US Medicare claims data, suggesting that even in regions with high survey-reported use, actual clinical implementation may be lower (19).

The pattern of barriers reported here suggests several distinct mechanisms rather than a single one. Concern about intracranial relapse and perceived insufficiency of the evidence are matters of clinical conviction, and are unlikely to respond to changes in resourcing; they point instead to a need for dissemination of the CC001 and RTOG 0614 results and of the reported hippocampal relapse rates. Resource-intensive planning, contouring time and treatment delay are operational constraints that could plausibly be addressed by automated or atlas-based hippocampal contouring and by protocolised planning pathways. Reimbursement and off-label status are system-level constraints outside the individual clinician’s control. Notably, reported availability of memantine within the respondent’s own centre was the strongest single correlate of both memantine and HA-WBRT offering in our exploratory models, and more than half of respondents did not know whether memantine was approved for this indication in their centre. This association is not evidence of a causal effect of availability itself: it may equally mark broader institutional readiness, including formulary processes, technical infrastructure and local awareness of cognitive-preservation strategies, particularly as it was also seen for HA-WBRT, a technique that does not depend on the drug. Read that way, formulary access and awareness of local approval status may be as important as clinical persuasion.

The infrequency of formal neurocognitive assessment in this cohort deserves comment in its own right. Only 27.4% of respondents reported using any cognitive assessment tool, and assessment was undertaken before radiotherapy and during follow-up in equal proportions. Without baseline and serial testing, clinicians have no local means of observing the neurocognitive decline that these interventions are designed to prevent, nor of recognising any benefit from adopting them. Brief, validated instruments suitable for routine oncology clinics exist, and their wider use would both improve individual patient care and generate the regional outcome data that this field currently lacks.

The substantial gap between guideline recommendations and real-world practice identified in this study has important clinical implications. The gap between reported offering and reported utilisation identifies a potential implementation gap. This study measured clinician-reported practice only; it did not measure patient-level treatment delivery or cognitive outcomes, and therefore cannot establish that any patient experienced avoidable neurocognitive decline. Improving access to cognitive-sparing techniques could meaningfully enhance quality of life for patients with an expected survival beyond four months.

Targeted educational initiatives addressing misconceptions about hippocampal relapse risk, alongside institutional support to streamline contouring and treatment planning workflows, may increase clinician confidence and adoption.

Memantine is orally administered, well tolerated and does not require the planning infrastructure that HA-WBRT demands, which makes it a plausible candidate for earlier implementation where HA-WBRT is not feasible. We did not collect data on price, reimbursement or access in the countries surveyed, and we therefore make no claim that it is uniformly low-cost across the region; the 46.8% of respondents who cited reimbursement difficulty suggests that cost and access are not uniformly favourable.

Future research should prioritise prospective, region-specific studies evaluating cognitive outcomes, quality of life, and cost-effectiveness of HA-WBRT and memantine in the MENA population. Implementation science approaches are needed to identify practical strategies for integrating neuroprotective techniques into high-volume clinical workflows.

Additionally, the development of standardised regional protocols, automated hippocampal contouring tools, and multidisciplinary training programmes may reduce technical barriers. Policy-level efforts addressing reimbursement and regulatory approval for memantine use in WBRT are also warranted. Further evidence is needed to clarify the role of HA-WBRT in subgroups currently excluded from major trials, including patients with small cell lung cancer and marginal performance status.

## 5 Limitations

This study has several important limitations, which we group under four headings.

### 5.1 Sampling and representativeness

Recruitment relied on open email and social-media circulation, so no sampling frame could be defined, no response denominator exists and no response rate can be calculated. The cohort is a convenience sample and is likely to over-represent clinicians with an existing interest in neuro-oncology, which would tend to inflate reported adoption. Saudi Arabia and Egypt together account for 60.5% of respondents, and ten of the fifteen countries contributed three or fewer respondents each, so no country-level inference other than the exploratory Saudi analysis is possible, and the findings should not be read as representative of the MENA region as a whole.

### 5.2 Clustering and independence

Multiple clinicians responded from the same institutions: among the 95 respondents who named a centre, 62 came from institutions contributing more than one response, and the largest contributed 11. Respondent-level answers are therefore not statistically independent. We addressed this with cluster-robust standard errors, but 29 respondents did not name an institution and had to be treated as singleton clusters, so the clustering correction is incomplete. Discordant answers from clinicians within the same institution were neither reconciled nor excluded.

### 5.3 Measurement

All outcomes are self-reported and are subject to social-desirability and recall bias; no patient-level or administrative validation was possible. The instrument was pilot-tested but not formally validated, and several response categories — particularly the distinction between offering an intervention, prescribing it and delivering it — are open to differing interpretation by respondents. Two items relating to memantine prescribing, and two relating to cognitive assessment, produced internally inconsistent answers, which we report as observed rather than reconcile.

### 5.4 Inference

The cross-sectional design cannot establish causation, temporal change or the direction of the associations observed. Many statistical comparisons were performed without adjustment for multiplicity, the Saudi subgroup analysis was *post hoc*, and several comparisons involved sparse cells. All inferential findings are accordingly exploratory and require confirmation.

## 6 Conclusion

To our knowledge, this is the first study to describe self-reported practice patterns of cognitive preservation during WBRT among radiation oncologists in the MENA region. Among the clinicians who responded, routine offering of HA-WBRT and memantine was limited, and reported delivery to patients was lower still.

Despite strong endorsement in international guidelines, routine adoption of HA-WBRT and memantine remains constrained by technical, experiential and systemic barriers. Targeted education, streamlined planning workflows and supportive policy frameworks are plausible routes to closing this gap. Whether underuse of these strategies extends across the MENA region as a whole cannot be determined from a convenience sample and would require a study with a defined sampling frame.

## Data Availability

The complete questionnaire is provided as Supplementary Material. The analysis code is available from the corresponding author on request. A deidentified respondent-level dataset is available on reasonable request, subject to removal or aggregation of country and centre identifiers for the smallest categories to minimize reidentification risk.
