# Supplementary material for: Self-reported practice patterns for cognitive preservation during whole-brain radiotherapy: a multinational cross-sectional survey of radiation oncologists in the Middle East and North Africa
Source: Front Oncol. 2026 Oct 13;16:1927397. doi: 10.3389/fonc.2026.1927397 (PMC13710059; doi:10.3389/fonc.2026.1927397)
Supplement: Supplementary file 1 [file DataSheet1.pdf]

Implementation of hippocampal avoidance and Memantine use with whole brain radiotherapy in solid tumor: A Clinical Survey.

1.

Thank you for participating in this survey.

This survey aims to gather information from radiation oncology professionals regarding the implementation of hippocampal avoidance (HA) technique and Memantine use with whole brain radiotherapy (WBRT).

Your participation will help identify current practices, challenges, and opportunities for improvement in these areas.

Your responses are anonymous and confidential, and the survey is voluntary. By continuing, you consent to participate and acknowledge that you understand the survey's purpose and conditions.

For any questions, please contact us at realhabib@moh.gov.sa.

Implementation of hippocampal avoidance and Memantine use with whole brain radiotherapy in solid tumor: A Clinical Survey.

2. Demographic data

1. Name (Optional):

[ ]

* 2. Gender:

- ○ Male
- ○ Female

3. Institution name:

[ ]

* 4. Institution country:

- ◯ Kingdom of Saudi Arabia
- ◯ United Arab Emirates
- ◯ Bahrain
- ◯ Oman
- ◯ Qatar
- ◯ Kuwait
- ◯ Egypt
- ◯ Jordan
- ◯ Tunisia
- ◯ Turkiye
- ◯ Other (please specify):

5. Institution city:

* 6. Years of independent practice:

- ◯ 1-5
- ◯ 6-10
- ◯ >10

* 7. Practice setting:

- ◯ University/Academic
- ◯ Private practice
- ◯ Public
- ◯ Military
- ◯ Other (please specify)

* 8. Do you treat adult Neuro-Oncological diseases?

- ◯ Yes
- ◯ No

Implementation of hippocampal avoidance and Memantine use with whole brain radiotherapy in solid tumor: A Clinical Survey.

3. Hippocampal avoidance whole brain radiotherapy (HA-WBRT)

Current practice of HA-WBRT

* 1. How many patients do you treat with brain metastasis annually?

- ○ <50
- ○ 50-100
- ○ 101-200
- ○ >200

* 2. How many patients do you treat with WBRT annually?

- ○ <50
- ○ 50-100
- ○ 101-200
- ○ >200

* 3. Which patients do you consider as not fit for HA-WBRT (Select all that apply)?

	Fit	Not fit
Poor performance status	○	○
Old age	○	○
Leptomeningeal disease	○	○
Patient with small cell lung cancer histology	○	○

* 4. How do you discuss the option of HA-WBRT with your patients ?

- ○ Routinely as a standard option of management
- ○ Only when specifically asked

4.

What are the main barriers for adopting HA-WBRT in your practice???

1. Time constraint related to contouring:

- ○ Yes
- ○ No

2. Difficulties with contouring:

- ◯ Yes
- ◯ No

3. Resource intense treatment planning:

- ◯ Yes
- ◯ No

4. Unavailability of the technique in my center:

- ◯ Yes
- ◯ No

5. Delay in starting the treatment:

- ◯ Yes
- ◯ No

6. Insufficient evidence support HA-WBRT:

- ◯ Yes
- ◯ No

7. No clinically relevant benefits:

- ◯ Yes
- ◯ No

8. Concern about missing area of the disease:

- ◯ Yes
- ◯ No

5.

Memantine use, Current practice

* 1. Is Memantine available in your center?

- ◯ Yes
- ◯ No

* 2. Is it approved for patients with brain metastasis?

- Yes
- No
- I don't know

* 3. How do you discuss the option of memantine with your patients?

- As standard of care of all patients with HA-WBRT
- Only when specifically asked

* 4. When do you start Memantine:

- As soon as I see the patient in clinic
- At the beginning of radiotherapy treatment
- After finishing WBRT
- I don't prescribe memantine

* 5. For how long do you prescribe Memantine?

- 3 months
- 6 months
- Without time limitations
- I don’t prescribe

Implementation of hippocampal avoidance and Memantine use with whole brain radiotherapy in solid tumor: A Clinical Survey.

6.

What are the main barriers to Memantine use?

* 1. Issues related to reimbursement due to off-label use:

- Yes
- No

* 2. Lack of experience with the drug:

- Yes
- No

* 3. Patient Compliance Issues:

- Yes
- No

* 4. Side effects and safety concerns:

- ◯ Yes
- ◯ No

* 5. Insufficient evidence support Memantine use:

- ◯ Yes
- ◯ No

* 6. No clinically relevant benefits:

- ◯ Yes
- ◯ No

Implementation of hippocampal avoidance and Memantine use with whole brain radiotherapy in solid tumor: A Clinical Survey.

7.

HA-WBRT & Memantine use, Current prcatice pattern

* 1. What percentage of your WBRT patients are treated with HA-WBRT + Memantine?

- ◯ Never
- ◯ <10%
- ◯ 10-25%
- ◯ 26-50%
- ◯ >50%

* 2. What percentage of your WBRT patients receive Memantine only?

- ◯ Never
- ◯ <10%
- ◯ 10-25%
- ◯ 26-50%
- ◯ >50

* 3. What percentage of your WBRT patients receive HA-WBRT only?

- ◯ Never
- ◯ <10%
- ◯ 10-25%
- ◯ 26-50%
- ◯ >50%

* 4. How frequent do you follow patients after HA-WBRT & Memantine use?

- ◯ Every 3 months for the first 1-2 years
- ◯ Every 6 months for the first 1-2 years
- ◯ Annually
- ◯ No follow up

* 5. What type of follow up do you use for patients treated with HA-WBRT & Memantine (Select all that apply)?

- ☐ Brain imaging
- ☐ Clinical assessment only
- ☐ Use of cognitive tests
- ☐ No follow up

* 6. What tests do you typically use for cognitive assessment?

- ◯ Mini Mental Status Test (MMST)
- ◯ Hopkins verbal learning test (HVLT)
- ◯ I don’t use any test
- ◯ Other (please specify)

7. When do we do the cognitive assessments?

- ◯ Prior radiotherpy
- ◯ Upon completion of radiotherpy
- ◯ During follow up
- ◯ I don't use cognitive tests

8. Please provide any additional thoughts or experiences related to HA-WBRT and Memantine:

Implementation of hippocampal avoidance and Memantine use with whole brain radiotherapy in solid tumor: A Clinical Survey.

8.

Thank you for your participation! Your insights are invaluable in understanding the current landscape of hippocampal sparing and Memantine use with whole brain radiotherapy in solid tumor.
